# Intervention to severe lower trachea obstruction supported by extracorporeal membrane oxygenation in a human immunodeficiency virus patient: A case report and literature review

**DOI:** 10.3389/fmed.2022.965721

**Published:** 2022-08-23

**Authors:** Xiaolin Zhang, Lei Pan, Lei Wang, Li Q. Li, Peng Zhang, Hai C. Tang, Qing G. Wu, Feng Li

**Affiliations:** ^1^Department of Respiratory Disease and Critical Care Medicine, Shanghai Public Health Clinical Center, Fudan University, Shanghai, China; ^2^Shanghai Institute of Infectious Disease and Biosecurity, Fudan University, Shanghai, China

**Keywords:** extracorporeal membrane oxygenation, trachea obstruction, interventional operation, HIV, trachea intubation

## Abstract

Here we reported a case, male, 33 years old, diagnosed with human immunodeficiency virus (HIV) infection 5 months ago, but he didn’t take antiretroviral drugs regularly. He was admitted to intensive care unit emergently due to hypoxemia, hypercapnia, and hypotension. CT showed severe lower trachea obstruction caused by soft tissue. After rapid bedside assessment, the patient was considered to need endotracheal operation, but he couldn‘t tolerate intubation and mechanical ventilation. Extracorporeal membrane oxygenation (ECMO) was used. Hemodynamics improved significantly along with rehydration and low-dose vasoactive drugs. Subsequently, the patient underwent rigid bronchoscopy, airway tumor resection and Y-type silicone stent implantation. Postoperatively protective endotracheal intubation and mechanical ventilation was followed. ECMO was weaned off after the operation, and endotracheal cannula was removed 6 h later. The pathological examination of excisional tissue showed lung squamous cell carcinoma. Finally, the patient was discharged safely and went to local hospital for further treatment. From this case, we conclude that ECMO could play a key role for those who need endotracheal surgery while cannot endure conventional intubation and mechanical ventilation.

## Case report

The patient, 33-year-old male, was admitted to Shanghai Public Health Clinical Center on November 22, 2019, with the complaint of “human immunodeficiency virus (HIV) infection for more than 5 months, cough and expectoration for 1 month, aggravation and shortness of breath for 2 days.” He was found HIV positive 5 months ago. There were no symptoms such as fever, cough, expectoration, nausea, vomiting, and he refused to accept any antiviral agents. Four month ago, he began to have anorexia and dysphagia. Gradually, he could only take liquid food and drink water. Two month ago, he began to accept antiretroviral drugs, but irregularly. Cough, yellowish white sticky sputum with blood in sputum occurred 1 month ago. Chest CT in local hospital showed that the right upper lung cavity was accompanied by multiple consolidation, and soft tissue density shadows in the trachea. He felt the above symptoms aggravated 2 days ago accompanied with severe shortness of breath, therefore he came to our hospital for further treatment and was admitted through Emergency.

Physical examination after admission found this patient extremely thin. With dyspnea gradually exacerbated, he received nasal catheter oxygen inhalation, transnasal high flow oxygen inhalation (HFNC) and non-invasive ventilator successively. On the 8th day after admission, the patient occurred obvious respiratory distress. Laboratory findings included WBC 12.63 × 10^9/L, HB 125 g/L, PLT 414 × 10^9/L, N 78.4%, L 13.1%, HS-CRP 65.4 mg/L, PCT 0.08 ng/ml, ALT 20 U/L, AST 18 U/L, TBIL 6.1 umol/L, ALB 36 g/L, K 3.7 mmol/L, CL 100 mmol/L, Na 138 mmol/L, UREA 2.95 mmol/L, Cr 49.5 umol/L, Glu 4.83 mmol/L, PT 13.7 S, APTT 41.8 S, FDP 7.1 μg/mL, D-D 2.76 μg/ml, TNI 0.01 ng/ml, pro-BNP 32 pg/ml, CK 40 U/L, HBsAg (-), anti-HCV (-), Anti-TP (-), T-SPOT.TB (-), CD4 195 cell/μl, CD8 597 cell/μl, CEA 0.87 ng/ml, CA125 122 U/ml, G test < 10 pg/ml, HIV 266 copy/ml (reference value < 40). Sputum smear found some coccus and bacillus, but culture was negative. His finger pulse oxygen saturation (SpO_2_) fluctuated obviously between 85 and 99%. Blood gas analysis showed pH 7.26, PaCO_2_ 9.6 kpa and lactic acid 7.65 mmol/L. He was supported by non-invasive ventilation with driving force of 14 cm H_2_O and inhaled oxygen concentration of 1.0 and transferred to ICU immediately.

**FIGURE 1:**
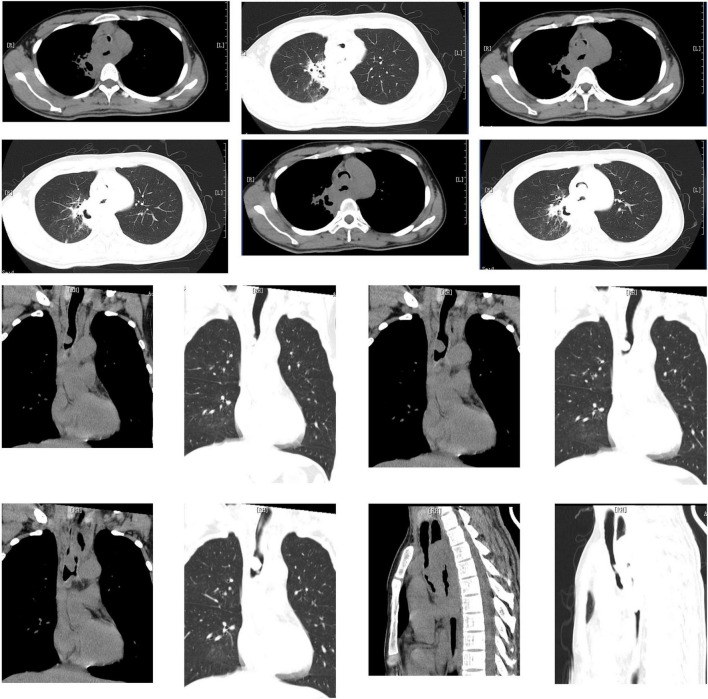
Cavitary lesions in the right upper lung, new organisms in the main trachea near to the carina which almost completely blocked the lumen (the narrowest part is about 2 mm), mediastinal lymph nodes swelling, obvious thickening of the wall of the middle esophagus and obvious expansion of the upper segment.

In order to clarify the cause of dyspnea, emergency CT was carried out and showed cavitary lesions in the right upper lung, new organisms in the main trachea near to the carina which almost completely blocked the lumen (the narrowest part is about 2 mm), mediastinal lymph nodes swelling, obvious thickening of the wall of the middle esophagus and obvious expansion of the upper segment (Figure 1). This revealed the patient’s dyspnea was due to severe stenosis of the trachea and he needed emergent airway operation. An emergent consultation of multiple disciplinary team (MDT) including anesthesia physician, intervention and ECMO team suggested that the patient’s vital signs were unstable, and the risk of preoperative anesthesia induction was very high. Meanwhile, due to airway severe obstruction, the conventional endotracheal intubation could not ensure sufficient ventilation. Extracorporeal membrane oxygenation (ECMO) was then considered to start. Bedside ultrasound evaluation found that the diameter of inferior vena cava was 13 mm, the variability 50%, the filling of right ventricle poor, and left ventricular systolic function normal. Fluid resuscitation was carried out and norepinephrine was continuously pumped intravenously (0.3 μg/kg/min) to maintain the stability of blood pressure.

After evaluation of blood vessels with ultrasound, we used Seldinger’s method to percutaneously puncture and put the catheters in the right femoral vein and right internal jugular vein. The tube sizes were Fr 21 and Fr 17, respectively. Initial setting of ECMO were rotating speed 3,200 rpm, blood flow 4.0 L/min and air flow 4.0 L/min (FiO_2_ 1.0). Low dose heparin was used for anticoagulation during catheterization, but anticoagulant agents were not given during ECMO running. With the oxygen supply from ECMO, the SpO_2_ increased rapidly from 75 to 100%. With the significant improvement of oxygenation, the real-time invasive arterial blood pressure stabilized at about 120/75 mmHg.

Next, we began to solve the airway obstruction. By inserting the rigid bronchoscope, we found that the neoplastic tissue in the lower part of the trachea lead to severe lumen stenosis. After circumcision of the tumor, a small amount of tissue infiltration could be seen at the opening of the left and right bronchia, and the distal lumen was basically unobstructed. After measuring the length of the neoplastic focus, a Y-shaped silicone stent was cut, sent to the carina, then released and adjusted. The fiberoptic bronchoscopy confirmed that the stent was stable and fit well with the tracheal wall. Withdrew the two scopes, followed by endotracheal intubation and mechanical ventilation. ECMO was weaned off when the operation finished and had been running for a total of 6 h. Ventilator and tracheal cannula were removed another 6 h later. The patient’s symptoms were significantly improved. Three days after the operation, bronchoscopy was carried out again and found the stent was in place and the lumen was normal. Pathological examination of excised tissue showed lung squamous cell carcinoma. Laboratory tests showed WBC 16.9 × 10^9/L, N 76.4%, L 15.3%, HS-CRP 95.3 mg/L, PCT 0.89 ng/ml. BALF culture for bacterial (-), fungal (-), tuberculosis (-), and he was treated with antibiotics, expectorants, and atomization therapy. The patient was discharged 1 week later and returned to local hospital for further treatment.

## Discussion

Some characteristics of immune system such as decreased CD4 + T lymphocyte counts and impaired natural killer cell (NK) function have been found in HIV patients, which could decline the ability of the body to monitor and kill tumor cells. The incidence of Kaposi sarcoma, non-Hodgkin lymphoma (NHL) and invasive cervical cancer show significantly higher in HIV positive patients (1, 2). With the application of antiretroviral therapy (ART), the survival time of HIV carriers has gradually prolonged, the type of tumor changed, and the proportion of non-AIDS defined cancer (NADC) gradually increased (3). The incidence of lung cancer in HIV is about 2–4 times that of non-HIV population, and the tumor is characterized by younger onset age, less typical pathological changes, and stronger invasion (4, 5). The malignant tissue in the airway can lead to obvious lumen stenosis. The swelling of mediastinal lymph nodes caused by tumor or opportunistic mycobacterial infection can worsen insufficient ventilation. Severe hypoxemia and carbon dioxide retention caused by major airway obstruction are life-threatening and usually require emergent disposition (6, 7).

Methods of respiratory support during airway intervention include nasal tube oxygen inhalation, face mask oxygen inhalation, high flow oxygen therapy, non-invasive ventilation, and endotracheal intubation to ensure oxygen supply. However, when severe stenosis occurs near the carina at the lower end of the trachea or bilateral main bronchus concurrently, the above methods might not be able to provide adequate ventilation, or even make it worse. In this situation, ECMO should be chosen to render sufficient oxygen, buy enough time for intervention surgery, and avoid ventilator-associated lung injury (8, 9). In 2015, Kim et al. reported 15 patients with upper respiratory tract obstruction due to various diseases received ECMO support during the airway intervention treatment. They suggested that ECMO should be considered when bronchoscopy or chest CT indicated the diameter of ventilation lumen was less than 5 mm (10).

In the present case, there was no attempt to intubate, which was the result of MDT discussion, based on the following reasons: 1. CT showed that the patient’s ventilation lumen was very small, about 2 mm (showed in lung window). In this case, intubation would reduce the ventilation space and interventional operation would further worsen it. 2. The patient was spontaneously breathing with assistance of non-invasive ventilator. The driving force of the ventilator was 14 cm H_2_O and inhaled oxygen concentration was 1.0. From the perspective of oxygen supply, intubation did not benefit the patient more. 3. If intubation and mechanical ventilation were performed, whether to keep the patient’s spontaneous breathing was a dilemma. In auxiliary mode with spontaneous breathing, intubation would significantly stimulate his irritability, leading to man-machine confrontation and increase of oxygen consumption. And in controlled mode, oxygen consumption would be reduced after the injection of analgesia and sedative drugs. But with the inhibition of spontaneous breathing, the ventilation of both dorsal parts was severely restricted. The imbalance of ventilation blood flow ratio might lead to further decline of oxygenation. Moreover, if intubation led to further deterioration of oxygenation, it would take an uncertain period of time to switch to ECMO. During this time, the patient was in extremely critical condition. If we chose ECMO, in the process of catheterization, we would not make the current situation worse without touching his airway.

Hong et al. reported that 19 patients with life-threatening hypoxemia caused by severe central airway obstruction underwent venous-venous (V-V) ECMO during intra-airway tumor removal and stent placement, which confirmed that ECMO could play a key role in lifesaving but very dangerous operations (11). Natt et al. reported a female patient who had acute respiratory failure due to severe centripetal stenosis under the cricoid cartilage after tracheotomy. Her physicians determined that the endotracheal intubation might not be successful. Therefore V-V ECMO was used, followed by bronchoscopic balloon dilatation and covered stent implantation. Then intubation and mechanical ventilation were implemented and ECMO was terminated (12). Malpas et al. reported a male patient suffering advanced thyroid cancer complicated with severe glottic stenosis so that tracheal access was difficult to establish. His physicians decided to start venous-arterial (V-A) ECMO under local anesthesia instead, then operated successfully (13).

In cases of airway obstruction caused by intraluminal neoplasm and airway compression caused by extraluminal tumor or lymphadenopathy, the intervention measures include rotary resection of intraluminal tumor or endotracheal stent implantation, or combination of both, which should be carried out based on rigid bronchoscope and high-frequency ventilation to ensure oxygenation (14). Commonly used airway built-in stents include silicone stents and self-expanding metal ones. Silicone stents can cover the fistula caused by malignant tumor, avoid restenosis caused by tumor recurrence, provide opportunities for chemotherapy or radiotherapy, and can be removed when the tumor shrinks after treatment (15).

Analgesic and sedative drugs can lead to breath depression, which could worsen hypoxemia of the patients with large airway obstruction. Local infiltration anesthesia with lidocaine during cannula insertion to keep the patient be awake and breathe autonomously can avoid further decline of oxygenation. Malpas et al. chose local infiltration anesthesia with lidocaine to start V-A ECMO after intravenous injection of midazolam (13). Kim et al. reported to use oxygen storage mask for preoxygenation and infuse remifentanil and dexmedetomidine intravenously for sedation (16). In the present case, we selected lidocaine for local anesthesia during catheterization, and intravenous injection of midazolam, sufentanil and muscle relaxant after ECMO was working. Before ECMO started, non-invasive ventilator was used to ensure oxygen supply with driving pressure of 15 cm H_2_O and oxygen concentration of 1.0. It should be noted that when the patient’s airway was severely obstructed, no matter what sort of supportive ventilation was used, there was a great risk. The most important thing was to insert the tube and start ECMO as soon as possible.

V-V ECMO was the primary choice while patients suffered from severe hypoxemia caused by critical lung disease. However, when hypotension existed at the same time, V-A ECMO should be considered as an option. In this critical scenario, whether the blood pressure could be controlled quickly became the key to the selection of ECMO mode. Although the patient we reported had shock, the results of rapid bedside assessment showed that his heart filling was not enough, and the systolic function was acceptable. We concluded that the cause of hypotension was partly due to acidosis caused by carbon dioxide retention and lactic acid accumulation, and partly due to insufficient circulating volume. So, the patient’s blood pressure could be better after fluid resuscitation and use of vasoactive drugs. Actually, after rapid fluid infusion and intravenous injection of low-dose norepinephrine, his blood pressure was under control. After ECMO started, the blood pressure was further stabilized while hypoxia and acidosis were corrected. However, it should be pointed out that V-A ECMO might be the better choice if the patient’s hypoxemia worsened and resulted in more severe shock. Sometimes, when it is uncertain whether V-V ECMO could successfully prevent further deterioration, it is necessary to be geared up for V-A ECMO. Zhang and colleagues placed a catheter in the left femoral artery for rapid conversion to V-A ECMO while inserting cannulas at the right femoral vein and the right internal jugular vein for V-V ECMO to treat a patient with relapsing polychondritis (17).

Anticoagulation is an important part of ECMO management, which is determined by the patient’s underlying diseases, coagulation state, the type of pipeline and oxygenator used, ECMO running time and blood flow velocity in the pipeline (18). During ECMO working, increasing blood flow velocity, and decreasing running time can significantly reduce the risk of thrombosis (19). In Hong et al. report, 13 cases received intravenous injection of heparin (3,000–5,000 IU) during blood vessel catheterization, and other 6 cases received nafamostat mesilate infusion. However, anticoagulation was not given during ECMO running in all 19 cases. In the end, 18 patients showed no complications such as bleeding or thrombosis and were discharged alive (11). These research suggested that ECMO could operate safely for a short time without anticoagulation. In the present case, low-dose heparin (50 IU/kg) was given intravenously during catheterization, while no anticoagulants were infused after ECMO started. After the operation, we followed up the coagulation function including D dimer and bedside ultrasound. No obvious bleeding or thrombosis were found during the whole hospitalization.

All in all, for most patients with hypoxemia due to airway obstruction, endotracheal intubation was the primary choice. V-V ECMO should be considered first only in the following cases: 1. The airway stenosis was so severe that the intubation itself had a high risk of exacerbation. 2. Endotracheal intubation had a high risk of failure, and at the same time there was no chance for tracheotomy for various reasons. 3. Patients had severe lung diseases, resulting in the inability of effective gas exchange even with endotracheal intubation, such as diffused pulmonary fibrosis, air leakage syndrome, pulmonary embolism, and extensive alveolar lesions. 4. Patients could tolerate endotracheal intubation, but could not endure endotracheal intervention, because interventional surgery itself might aggravate hypoxia. Preoperative evaluation should be carried out comprehensively to decide ECMO operation mode. The most important thing is to put in the tubes and start the machine at the top speed, and it is safe not to use anticoagulation for a short time. Certainly, patients without systemic anticoagulation should be followed up with coagulation function and ultrasound to assess the risk of thrombosis and bleeding. Weaning ECMO off quickly after the surgery can reduce ECMO related complications after the transition to conventional mechanical ventilation.

## Data availability statement

The raw data supporting the conclusions of this article will be made available by the authors, without undue reservation.

## Author contributions

XZ, LP, and LW: manuscript writing and results discussion. LL, PZ, and HT: data curation, review, and study supervision. QW and FL: conception and design, formal analysis, and manuscript revision. All authors contributed to the article and approved the submitted version.
